# Genomic and epigenomic variation in *Psidium* species and their outcome under the yield and composition of essential oils

**DOI:** 10.1038/s41598-023-27912-w

**Published:** 2023-01-25

**Authors:** Matheus Alves Silva, Fernanda Aparecida Ferrari Soares, Wellington Ronildo Clarindo, Luiza Alves Mendes, Luziane Brandão Alves, Adésio Ferreira, Marcia Flores da Silva Ferreira

**Affiliations:** 1 Departamento de Agronomia, Centro de Ciências Agrárias e Engenharias, Universidade Federal do Espírito Santo, Alto Universitário, s/n, Guararema, Alegre, ES 29500-000 Brazil; 2 Departamento de Biologia Geral, Universidade Federal de Viçosa, Av. Peter Henry Rolfs, s/n, Campus Universitário, Viçosa, MG 36570-900 Brazil; 3 Departamento de Química, Universidade Federal de Viçosa, Av. Peter Henry Rolfs, s/n, Campus Universitário, Viçosa, MG 36570-900 Brazil

**Keywords:** Genetics, Agricultural genetics, Cytogenetics, Epigenetics, Plant genetics

## Abstract

Diploid and polyploid species derived from the euploid series x = 11 occur in the genus *Psidium*, as well as intraspecific cytotypes. Euploidy in the genus can alter the gene copy number, resulting in several “omics” variations. We revisited the euploidy, reported genomic (nuclear 2C value, GC%, and copy number of secondary metabolism genes) and epigenomic (5-mC%) differences in *Psidium*, and related them to essential oil yield and composition. Mean 2C values ranged from 0.90 pg *(P. guajava*) to 7.40 pg (*P. gaudichaudianum*). 2C value is intraspecifically varied in *P. cattleyanum* and *P. gaudichaudianum*, evidencing cytotypes that can be formed from euploid (non-reduced) and/or aneuploid reproductive cells. GC% ranged from 34.33% (*P. guineense*) to 48.95% (*P. myrtoides*), and intraspecific variations occurred even for species without 2C value intraspecific variation. Essential oil yield increased in relation to 2C value and to GC%. We showed that *P. guajava* (diploid) possesses two and *P. guineense* (tetraploid) four copies of the one specific TPS gene, as well as eight and sixteen copies respectively of the conserved regions that occur in eight TPS genes. We provide a wide “omics'' characterization of *Psidium* and show the outcome of the genome and epigenome variation in secondary metabolism.

## Introduction

The neotropical and monophyletic genus *Psidium*, family Myrtaceae, contains approximately 92 species^1^, hich are taxonomically classified in four sections: *Psidium* (10 species), *Obversifolia* (six species), *Apertiflora* (31 species) and *Mitranthes* (26 species)^2^. ~ 60 *Psidium* species occur in Brazil distributed in all biomes, in several phytogeographic domains, representing great diversification^3^. *Psidium guajava* L. (guava tree, *Psidium* section) is the well-known species of the genus due to its relevance for fruit production^4^ and medicinal value^5,6^. Other *Psidium* species, popularly called “araçás”, are potential genetic resources for breeding programs and medicinal purposes^7^, as well as they are relevant for taxonomic, ecological and evolutive studies in this genus^2^.

Besides diploid species, in *Psidium* occur polyploid species with different ploidy levels commonly derived from the basic chromosome number x = 11. One of the consequences of euploidy in *Psidium* is the interspecific and intraspecific variation of the nuclear 2C value. Previously, we found that the increase (2C = 0.93 pg – 2C = 5.12 pg) of the nuclear 2C value is outcome of the increase in ploidy level (diploid with 2C = 0.93 pg – 2C = 0.98 pg to octoploid with 2C = 5.12 pg) in seven *Psidium* species^8^. In addition to interspecific variation, genomic differences have been identified for *P. cattleyanum*. This species is considered a polyploid complex due to genetic diversity (intraspecific variation) of the 2n chromosome number and, consequently, the ploidy level (triploid 2n = 3x = 33 to duodecaploid 2n = 12x = 132 chromosomes), nuclear 2C value (tetraploid with 2C = 2.17 pg to duodecaploid with 2C = 5.64 pg), and number of the CMA3 + /DAPI-, 18S rDNA and 5S rDNA sites (triploid with three sites to duodecaploid with 12, 12 and 10 sites)^9^.

Despite the 2n chromosome number importance for ploidy level determination (euploidy condition), previous data indicate that the nuclear 2C value is an indicator of higher ploidy level in *Psidium*^8,9^. Thus, nuclear 2C value can be used especially when dealing with a large number of individuals. Nuclear 2C value is mainly measured using flow cytometry, which is widely used because it is fast, accurate and reproducible^10^.

In addition to the nuclear 2C value, the AT/GC base composition can also be measured by flow cytometry, expanding the genomic data^11^. AT/GC base composition knowledge allows inferences about the genome structure and dynamic. So, AT/GC base composition must be incorporated into “omics” data, and consequently used in taxonomic, systematic and evolutive studies. *Psidium guajava* genome has GC = 39.5%^12^, and *P. cattleyanum* transcriptome has ~ 49% in the yellow and red morphotypes^13^.

Polyploidy has been considered one of the main genomic changes that results in genetic^8^ and epigenetic modifications^14^ influencing the population genetic structure, ecological niche differentiation, diversification and speciation in plants^15^. About it, *Psidium* is one outstanding example of the polyploid impact in speciation and geographic distribution^8,15^. Euploidy (autopolyploidy, true allopolyploidy or segmental allopolyploidy) plays a central role in shaping and restructuring plant genomes^15^. Regardless of the genomic origin, one euploidy outcome is the increase of the gene copy number, which probably results in phenotypic changes and/or new traits. Also furthering phenotypic variations, genomic changes occur (“genomic shock”) after the euploidy, such as aneuploidy, structural chromosome rearrangements, mobile elements activation or silencing, and DNA sequence change^16^. Thus, the euploidy and its outcomes are sources of evolutionary novelties. Duplicate genes can follow an evolutive path from an initial state of complete redundancy, in which a copy is probably disposable, to a stable situation^17,18^ – neutral theory. Still, they may include new gene functions and expression patterns. The duplicated genes can maintain their original or similar function, undergo diversification in function or expression patterns, or a copy can be silenced by mutations or epigenetic mechanisms^19^. Many duplicate genes can have *loci* in tandem in the genome or occur regionalized (within a few Mbp)^20,21^. Additionally, phylogenetic evidence links genome increase (nuclear 2C value) to the increase in the overall percentage of global 5-methylcytosine (5-mC%). 5-mC is an important epigenetic chemical change in DNA that promotes heterochromatinization (chromatin compact level increase) and, consequently, gene expression control^22,23^.

Leaf essential oils, characteristic of the Myrtaceae family secondary metabolism, are rich in terpene and exhibit quali- and quantitative variations reported^24^. Terpenes play ecological roles, and essential oils are economically exploited^25–27^ for their biological and phytotherapeutic activities^28^. Our research group evidenced that seven *P. cattleyanum* plants exhibited different nuclear 2C values (2C = 3.20 pg – 2C = 6.03 pg), seven monoterpenes and eight sesquiterpenes in essential oils. From these data, the *P. cattleyanum* plants were discriminated in three cytotypes (nuclear 2C value) related to three chemotypes (monoterpene and sesquiterpene compounds). *P. cattleyanum* plants with the relatively lower nuclear 2C values (2C = 3.23 pg – 2C = 4.71 pg) produced a lower amount of essential oils composed mostly of hydrogenated monoterpenes. Differently, the plants with relatively higher nuclear 2C values (2C = 5.81 pg and 2C = 6.03 pg) produced a higher amount of essential oils composed mostly of hydrogenated sesquiterpenes, as trans-caryophyllene and alpha-humulene^24^.

We aimed to revisit the 2n chromosome number (euploidy), measure the nuclear 2C value, GC% and 5-mC%, and determine the copy number of TPS genes in *Psidium* species of three sections (*Psidium*, *Apertiflora* and *Obversifolia*). In addition, we correlated these genomic and epigenomic data with the yield and composition of the essential oils, evidencing the outcomes of the genetic and epigenetic differences in this phenotype.

## Results

### Intraspecific and interspecific variation of nuclear 2C value and GC%

The nuclear 2C value of each *Psidium* access ranged from 0.90 pg (*P. guajava*) to 7.40 pg (*P. gaudichaudianum*, Supplementary Table S2). We noticed, for the first time, the nuclear 2C value for *P. acidum*, *P. gaudichaudianum*, *P. friedrichsthalianum*, *P. macahense* and *P. rufum*. *Psidium guajava*, *P. oblongatum* and *P. macahense* presented the lowest 2C values. *Psidium guajava* and *P. oblongatum* are diploids (2n = 2x = 22 chromosomes). Therefore, possibly *P. macahense* also has the same 2n chromosome number and ploidy level, since this species has closer mean nuclear 2C value (2C = 0.93 pg) than *P. guajava* (2C = 0.96 pg) and *P. oblongatum* (2C = 0.99 pg).

Interspecific variation in nuclear genome size was confirmed by mean nuclear 2C values of the *Psidium* species (Table 2). Considering the lowest mean 2C value = 0.93 pg for *P. macahense* and highest 2C = 4.99 pg for *P. gaudichaudianum*, we realize a variation equivalent to 2C = 4.06 pg more nuclear DNA. Additionally, the individual nuclear 2C values also show the interspecific variation in nuclear genome size, reaching 2C = 6.50 pg more nuclear DNA, since one access of *P. guajava* has 2C = 0.90 pg and one access of *P. gaudichaudianum* has 2C = 7.40 pg.

In addition to interspecific variation, the individual values point to intraspecific variation of the nuclear 2C value, including among relatives. Accesses belonging to *P. guajava* (2C difference = 0.13 pg among individuals), *P. guineense* (2C = 0.20 pg) and *P. acidum* (2C = 0.08 pg) showed less variation of the nuclear 2C value. These nuclear 2C values differences are lower than the 1Cx value of *P. guajava* (0.475 pg) and *P. oblongatum* (0.490 pg) determined considering the basic chromosome number (x = 11) of the genus *Psidium* and the ploidy level of these species (2n = 2x = 22 chromosomes – diploid^8^). Therefore, the intraspecific variation found among accesses of *P. guajava*, *P. guineense* and *P. acidum* is probably a consequence of secondary metabolites that interfere with the intercalation of the propidium iodide fluorochrome to DNA in the staining step for nuclear suspension preparation for flow cytometry.

We also observed intraspecific variation of the nuclear 2C value among individuals, as well as in the relatives of *P. myrtoides*, *P. cattleyanum* and *P. gaudichaudianum*. The nuclear 2C value difference was: 2C = 0.40 pg between individuals of *P. myrtoides*, 2C = 5.03 pg of *P. cattleyanum*, and 2C = 2.76 pg of *P. gaudichaudianum*. For these species, the nuclear 2C value differences are close to or higher than the reference 1Cx value (1Cx = 0.475 pg *P. guajava*—1Cx = 0.490 pg *P. oblongatum*) at the basic chromosome number x = 11 of *Psidium*. Therefore, these values indicate that individuals of these species have different 2n chromosome numbers among each other, possibly arising from numerical chromosomal changes (euploidy and/or aneuploidy).

According to the mean nuclear 2C values (Table 2) and the data reported for diploid and polyploid *Psidium* species (Table 1, http://ccdb.tau.ac.il/search/, https://cvalues.science.kew.org/search/angiosperm), we suggest that accesses of the species *P. acidum, P. rufum, P. friedrichsthalianum* and *P. gaudichaudianum* are potential polyploids. Thus, individuals of these species probably have more than 2n = 2x = 22 chromosomes. However, chromosome number counting should be conducted to confirm the ploidy level.

**Table 1:** Section, habit, Brazilian region of occurrence, phytogeographic domain, chromosome number and ploidy level reported for *Psidium* species.

Specie and Section^2^	Habit^3^	Occurrence^3^	Phytogeographic domain^3^	Chromosome number and ploidy level
*Psidium acidum* (DC.) Landrum (section *Psidium*)	Tree	North, Southeast, South	Amazon	–
*P. cattleyanum* Sabine (section *Obversifolia*)	Tree	Northeast, Southeast, South	Caatinga, Cerrado, Atlantic Forest	46, 55, 58 and 82^29^; 33 (3x), 44 (4x), 55 (5x), 66 (6x), 77 (7x), 88 (8x), 99 (9x), 110 (10x) and 132 (12x)^9^
*P. guajava* L. (section *Psidium*)	Tree	North, Northeast, Center-West, Southeast, South	Amazon, Caatinga, Cerrado, Atlantic Forest	22 (2x)^29,30^
*P. guajava* L. x *P. guineense* Sw	–	–	–	–
*P. guineense* Sw. (section *Psidium*)	Shrub, Tree	North, Northeast, Center-West, Southeast, South	Amazon, Caatinga, Cerrado, Atlantic Forest	44 (4x)^30^
*P. myrtoides* O.Berg (section *Apertiflora*)	Tree	North, Northeast, Center-West, Southeast, South	Caatinga, Cerrado, Atlantic Forest	66 (6x)^8^; 88 (8x)^31^
*P. gaudichaudianum* Proença & Faria	Tree	Southeast	Atlantic Forest	-
*P. friedrichsthalianum* (O.Berg) Nied (section *Psidium*)	Shrub, Tree	North	Amazon	44 (4x)^31^
*P. macahense* O.Berg (section *Apertiflora*)	Shrub	Southeast	Atlantic Forest	-
*P. oblongatum* O.Berg	Tree	Southeast	Atlantic Forest	22 (2x)^8^
*P. rufum* Mart. ex DC. (section *Apertiflora*)	Tree	Northeast, Center-West, Southeast, South	Cerrado, Atlantic Forest	–
*Psidium* sp.	–	–	–	–

Six groups were identified in relation to the individual nuclear 2C value (Table 3). Group I comprises the individuals with the smallest nuclear 2C value (2C = 0.90 pg to 2C = 1.10 pg), which include the diploid species *P. guajava, P. oblongatum*, and the *P. machaense* that, based on the 2C and 1Cx values, possible is a diploid species. Group II (2C = 1.80 pg—2C = 2.08 pg) has the tetraploid species *P. guineense* and *P. acidum*, as well as all 14 *P. guajava* × *P. guineense* hybrids, one individual of *P. cattleyanum* and seven individuals of *Psidium sp*. The evaluated *P. guajava* × *P. guineense* hybrids have 2C = 1.90 pg, exhibiting the same nuclear 2C value (Table 2). This mean value is equivalent to the tetraploid genomic origin. Different progenies are possible considering the *P. guajava* × *P. guineense* crossing: (a) allotriploid hybrids (~ 2C = 1.43 pg) from the fusion of reduced reproductive cells of the two species, (b) allotetraploid hybrids (~ 2C = 1.91 pg) generated from the fusion of non-reduced reproductive cells of *P. guajava* and reduced *P. guineense*, (c) allopentaploid hybrids (~ 2C = 2.38 pg) formed by fusing reduced reproductive cells of *P. guajava* and non-reduced *P. guineense*, and (d) allohexaploid hybrids (~ 2C = 2.85 pg) arising from the fusion of non-reduced reproductive cells of the two species. Therefore, probably the *P. guajava* × *P. guineense* hybrids with 2C = 1.90 pg are the result of the fusion of non-reduced reproductive cells of *P. guajava* and reduced *P. guineense*. In addition, we found a *P. guajava* × *P. guineense* hybrid (Hib_11) mixoploid with 30% of cells with 2C = 0.95 pg (similar to diploid *P. guajava*) and 70% of cells with 2C = 1.90 pg (similar to tetraploid *P. guineense*) (Supplementary Table S2).

**Table 2:** Nuclear DNA content (2C value) and percentage of GC bases (GC%) of *Psidium* accesses and half-sibling family (MI).

Access	Valor 2C (pg)		CG%	
	Mean ± SD	Minimum and maximum value	Mean ± SD	Minimum and maximum value
*P. macahense*	0.93	–	37.56	–
*P. guajava*	0.96 ± 0.030	0.90–1.10	37.97 ± 1.092	36.16–40.74
*P. oblongatum*	0.99	–	39.81	–
*P. guajava x P. guineense*	1.90 ± 0.000	1.90–1.90	35.64 ± 0.506	35.07–36.08
*P. guineense*	1.90 ± 0.078	1.80–2.00	36.86 ± 3.535	34.33–43.07
*P. acidum*	2.04 ± 0.043	2.00–2.08	40.29 ± 0.356	40.04–40.54
*P. myrtoides*	2.95 ± 0.090	2.72–3.12	39.62 ± 2.015	37.63–48.95
*P. cattleyanum*	3.92 ± 0.618	2.00–7.03	40.40 ± 2.815	36.37–48.91
*P. rufum*	4.23	–	–	–
*Psidium* sp.	4.70 ± 0.411	1.92–4.94	38.98 ± 1.858	35.90–40.92
*P. friedrichsthalianum*	4.72	–	38.59	–
*P. gaudichaudianum*	4.99 ± 0.477	4.64–7.40	39.67 ± 0.723	38.38–40.48
*P. guajava-*MI 06	0.96 ± 0.022	0.92–1.00	37.91 ± 0.899	36.27–39.29
*P. guajava-*MI 05	0.97 ± 0.033	0.95–1.03	38.59 ± 1.140	37.32–40.33
*P. myrtoides-*MI 08	2.93 ± 0.099	2.72–3.12	43.83 ± 0.599	38.71–48.95
*P. myrtoides-*MI 07	3.00 ± 0.060	2.82–3.09	39.38 ± 0.900	38.37–40.49
*P. cattleyanum-*MI 03	3.67 ± 0.195	3.34–4.27	39.94 ± 1.295	37.99–41.46
*P. cattleyanum-*MI 02	3.82 ± 0.215	3.45–3.98	–	–
*P. cattleyanum-*MI 01	3.86 ± 0.663	3.29–5.68	–	–
*P. cattleyanum-*MI 04	4.16 ± 0.161	3.63–4.38	41.91 ± 0.962	41.23–42.59
*P. gaudichaudianum-*MI 09	4.99 ± 0.485	4.64–7.40	39.72 ± 0.800	38.38–40.48

Group III (2C = 2.72—2C = 4.38 pg) contains most of the individuals evaluated, including the species *P. cattleyanum, P. myrtoides, P. rufum* and one *Psidium sp*. Due to the variation in 2n chromosome number and ploidy level of *P. cattleyanum* and *P. myrtoides* (Table 1), the group III includes possibly hexaploid and octaploid accesses. Group IV (2C = 4.47 pg—2C = 5.19 pg) has individuals from *P. cattleyanum, P. gaudichaudianum, P. friedrichsthalianum* and three *Psidium sp*, which are probably octaploids (Table 1), as well as the only individual from group V of *P. cattleyanum*. Group VI includes the three individuals with the highest 2C values (2C = 6.83 pg—2C = 7.40 pg), two from *P. cattleyanum* and one from *P. gaudichaudianum*. Probably the accesses in group VI have a ploidy level higher than octaploid (2n = 8x = 88 chromosomes).

*Psidium cattleyanum* showed the highest intraspecific variation of the nuclear 2C value, exhibiting individuals in five of the six groups. Differently, less intraspecific variation was confirmed for the diploid species *P. guajava* (all individuals in group I), for the tetraploids *P. guineense* and *P. acidum* (group II) and for the hexaploid *P. myrtoides* (group III, Table 3).

**Table 3:**
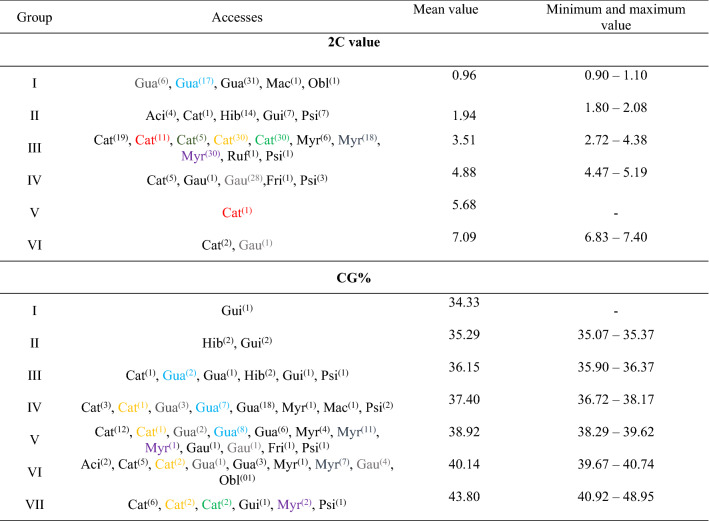
Grouping by Tocher’s method (optimized) of nuclear DNA content (2C value) (pg) and percentage of GC bases (GC%) of *Psidium* accesses.

Aci = *P. acidum*; Cat = *P. cattleyanum*; Gua = *P. guajava*; Hib = *P. guajava x P. guineense*; Gui = *P. guineense*; Myr = *P. myrtoides*; Gau = *P. gaudichaudianum*; Fri = *P. friedrichsthalianum*; Mac = *P. macaense*; Obl = *P. oblongatum*; Ruf = *P. rufum*; Psi = *Psidium* sp. Superscript value in parentheses = number of individuals of the species present in the group. Color representations = Half-sib families  (separately accounted).

The GC% values of the evaluated 137 individuals ranged from 34.33% for an individual of *P. guineense* to 48.95% for an individual of *P. myrtoides.* The largest intraspecific variations (~ 10%) were in *P. myrtoides* and *P. cattleyanum*, which are species that also show nuclear 2C value intraspecific variation. For the other species, the GC% ranged from 0.50% for *P. acidum* to 8.74% for *P. guineense*. The variation in the two half-sib families of *P. myrtoides* was 2.12% (Family 7) and 10.24% (Family 8). Within families of *P. cattleyanum*, a small variation was observed (1.36% for Family 7 and 3.47% for Family 3), despite intraspecific nuclear genome size variation in the species of up to 2C = 5.03 pg. The intraspecific variation of GC% was ~ 3.00% for the two families of *P. guajava*, and of ~ 2.10% *P. gaudichaudianum* (Table 3).

Seven groups were obtained from comparative GC% analysis. *Psidium cattleyanum, P. guajava, P. guineense, P. myrtoides, P. gaudichaudianum, P. guajava* × *P. guineense* hybrids, and *Psidium sp*. showed individuals in at least two groups, evidencing GC% intraspecific variation. Groups III—VI consisted of diploid and polyploid species. Only one family of *P. cattleyanum* showed greater stability being allocated only to group VII (Table 3).

### 5-mC%, yield and chemical composition of the essential oil

We compiled in Supplementary Table S3 the unpublished and published values, for each *Psidium* individual, of 5-mC%, yield, and the percentage of the chemical compounds identified from the essential oil. 5-mC values varied between 16.34% (*P. guajava*) to 33.30% (*P. myrtoides*), and between 0.20% (*P. guajava*) to 0.95% (*P. cattleyanum*) for yield of the essential oil. A total of 56 compounds were identified for *Psidium* species. Of these compounds, 55 are chemically classified between hydrocarbons and oxygenated mono- and sesquiterpenes.

### Relationship of the mean nuclear 2C value, GC% and 5-mC% values, yield and chemical composition of the essential oil

The nuclear 2C and GC% values were positively correlated (correlation of 0.51). These values also had positive correlation with 5-mC% (correlation of 0.35 and 0.36, respectively) and with essential oil yield (correlation of 0.72 and 0.70, respectively). The variables related to genome (nuclear 2C value and GC%) and to epigenome (5-mC%) correlated negatively with (E)-Nerolidol (correlation of − 0.64, − 0.45 and − 0.55, respectively), β-Bisabolol (correlation of − 0.61, − 0.46 and − 0.35, respectively) and the group of oxygenated sesquiterpenes (− 0.87, − 0.70 and − 0.46, respectively). The α-Pinene and the hydrocarbon monoterpene group were positively correlated with nuclear 2C value (correlation of 0.54 and 0.49, respectively) and 5-mC% (correlation of 0.60 and 0.53, respectively). The nuclear 2C value along with GC% correlated positively with the β-caryophyllene (correlation of 0.70 and 0.65, respectively), α-Copaene (correlation of 0.57 and 0.56, respectively) and the hydrocarbon sesquiterpene group (correlation of 0.42 and 0.52, respectively). These genomic data correlated negatively with 14-Hydroxy-epi-(E)-Caryophyllene (correlation of − 0.50 and − 0.46, respectively) and Selin-11-en-4a-ol (correlation − 0.44 and − 0.38, respectively), which belong the oxigenated sesquiterpene group. In addition, the β-Selinene and Hinesol correlated negatively with nuclear 2C value (correlation of − 0.35 and − 0.42 respectively).

### TPS gene copy number

**Figure 1:**
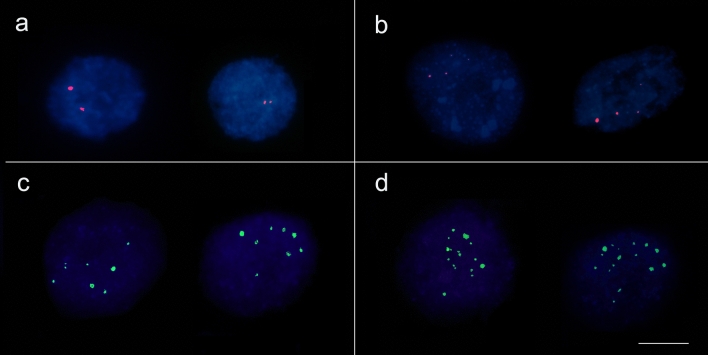
Copy number of TPS genes. *P. guajava* possesses one copy of the specific TPS gene **(a)**, while *P. guineense* shows two copies of this gene **(b)**. From the probe in conserved motifs, we evidenced eight fluorescence signals in *P. guajava* **(c)** and sixteen in *P. guineense* **(d)**, being that strong fluorescence signals are related to cluster genes. Based on these results, we showed the polyploidy impact in the gene copy number in *Psidium*.

Copy number of TPS genes was determined in diploid *P. guajava* (2n = 2x = 22 chromosomes and 2C = 0.95 pg) and tetraploid *P. guineense* (2n = 4x = 44 chromosomes and 2C = 1.90 pg). We expected the copy number to be exactly double in the tetraploid *P. guineense*. The genes showed distinct marking patterns when comparing the hybridization signal number in relation to the ploidy of the species. For the specific region used, we detected two hybridization signals in *P. guajava* and four in *P. guineense* nuclei (Fig. 1).

From the general primer of the conserved motives, the *P. guajava* nuclei showed four strong signals and four weak signals. *P. guineense* nuclei exhibited eight strong and eight weak signals (Fig. 1). The presence of weak signals was considered as DNA sequences that have a relatively homology in relation to the probe, corresponding to regions that had the same origin, but that accumulated differences in the gene sequence. In addition, the weak hybridization signals can be resulted by occurrence of a single gene copy. Differently, the strong hybridization signals correspond to gene copies in tandem repeats, which form clusters that amplify the fluorescence signal.

Therefore, we confirmed that the copy number of the TPS genes was directly related to the ploidy level of the species with double of signals found in the tetraploid *P. guineense*. This result also shows the genome evolution of these *Psidium* species.

## Discussion

We report the nuclear 2C and GC% values, TPS gene copy number, as well as 5-mC% and essential oil yield and composition of a considerable number of *Psidium* species and individuals**.** The GC% values, with the exception of *P. guajava*^12^, are unpublished for all *Psidium*, as well as the nuclear 2C values for the *P. acidum, P. gaudichaudianum, P. friedrichsthalianum, P. macahense* and interspecific hybrids (*P. guajava L.* × *P. guineense*). 5-mC% are also unpublished for *P. cattleyanum, P. guineense, P. myrtoides, P. gaudichaudianum, P. friedrichsthalianum, P. oblongatum* and one access of the genus. In addition, the determination of the TPS gene copy number evidenced other genomic outcomes of the polyploidy beyond the nuclear 2C and GC% interspecific variation. The results bring advances about the structure, organization and evolution of the genome and epigenome of *Psidium* species in inter- and intraspecific contexts, including analyses of related individuals. The genomic and epigenomic data were contextualized with the yield and diversity of compounds in the leaf essential oils, which are rich in mono- and sesquiterpenes that have ecological and economic importance for the Myrtaceae family.

Based on the previous study of the our research group and the basic chromosome set of *Psidium* (x = 11), the 1C value varied from 0.465 pg (*P. cauliforum*—diploid species) to 0.640 pg (*P. longipetiolatum*—octaploid species), and the increase in ploidy culminated in the increase in nuclear DNA content^8^. Intraspecific variation in 2C value has been demonstrated for *P. cattleyanum* (2C = 2.00 to 2C = 7.03 pg), corroborating reports of naturally occurring individuals with varied chromosome numbers and ploidy levels for the species, which has cytotypes with 2n = 46, 55, 58 and 82^29^; 33 (3x), 44 (4x), 55 (5x), 66 (6x), 77 (7x), 88 (8x), 99 (9x), 110 (10x) and 132 (12x)^9^ (Table 1). Thus, the 2n chromosome number variation in this species can also be supported by the 2C nuclear value variation. Furthermore, we infer that euploidy occurs in the genus not only in *P. cattleyanum*, but also in *P. gaudichaudianum* and *P. friedrichsthalianum* due to the amplitude of 2C nuclear value variation.

The oosphere and the reproductive nucleus of the pollen grain are usually reduced reproductive cells (n—haploid). However, non-reduced and/ou aneuploid reproductive cells can be formed due to errors in anaphase I or II during meiosis, by the non-disjunction of chromosomes^32^, and/or by the non-occurrence of cytokinesis I or II. Thus, reproductive cells with different euploidy and/or aneuploidy can be generated. Individuals of *P. cattleyanum* and *P. gaudichaudianum*, including relatives, showed expressive variations in nuclear 2C value (possibly reflecting the 2n chromosome number) that may have resulted from the unilateral or bilateral fusion of reduced and unreduced reproductive cells. The unreduced reproductive cells may come from one (unilateral) or both parents (bilateral) in cross-fertilization or self-fertilization^33^. In addition, numerical chromosomal variations (euploidy and aneuploidy) can occur in cells of meristematic regions resulting in mixoploid tissues and/or individuals. Thus, the male and/or female reproductive organs of the flowers can have meiocytes with different chromosome numbers compared to the sporophyte. In this context, it is important to highlight the occurrence of a mixoploid individual (Hib_11), not yet reported in *Psidium*. The mixoploidy can compromise the stability and fertility of plants in the field and, thus, the use of these plants for breeding purposes is not very desirable^34^.

In general, polyploid species of *Psidium* present a greater geographical distribution compared to diploids^8^, with the exception of *P. guajava* because it is widely exploited and cultivated and, therefore, present in the most varied regions and biomes^35^. This fact was pointed out by our research group, considering species *P. guajava, P. guineense, P. myrtoides, P. cattleyanum, P. longipetiolatum, P. oblongatum* and *P. cauliflorum*^8^. In addition, the geographical distribution of *P. cattleyanum* cytotypes is influenced by the ploidy level. *P. cattleyanum* cytotypes with higher ploidy levels were identified in regions where the environmental conditions are more adverse, with higher temperatures, higher incidence of solar radiation and lower precipitation^9,15,36^. Therefore, the polyploid condition of the species studied here, may be favorable for expansion of their geographic distribution, both by natural and anthropic action. Hence, exploitation and utilization of these natural resources is relevant for breeding programs and for familiar production.

We verified inter- and intraspecific variations of GC% for the diploid and polyploid species. Although variations occurred, the overall mean 38.92% CG in *Psidium* is close to the mean value of 38.06% obtained by means of 22 diploid *Eucalyptus* species and three of the genus *Corymbia*, also species of the Myrtaceae family^37^.

Due to 2C value and GC% variations in *Psidium*, especially in families, and their influence on secondary metabolism, we suggest, in a practical context, the individual pre-selection of plants to compose an experimental project, breeding program, germplasm bank or cultivation. In this sense, we recommended that the pre-selected accesses or individuals of *Psidium* should be vegetatively propagated, generating new individuals with the same 2n chromosome number, 2C nuclear value and GC% (genomic stability). On the other hand, inter- and intraspecific genomic diversity is important as a source of genetic resources for breeding.

We verified an increase in essential oil yield in *Psidium* due to the larger genome, evidencing the impact of the genomic changes (2n chromosome number and 2C nuclear value) in the secondary metabolism, which is a trait of ecological and economic importance. Experimentally, the tetraploid induction (2n = 4x = 72 chromosomes) in *Lippia integrifolia* (family Verbenaceae) increases the essential oil yield compared to diploids (2n = 2x = 36 chromosomes), in addition to larger leaves and trichomes, structures related to essential oil yield^38^. Additionally, we showed by FISH that the polyploidy increases the copy number of the orthologs of two TPS genes related to essential oil biosynthesis of *Psidium* species. Therefore, the polyploidy, also evidenced by 2C nuclear value, affects the essential oil yield in *Psidium* from the diploid species (*P. guajava* 2n = 2x = 22 chromosomes) and the hitherto reported closest species *P. guineense* (2n = 2x = 44). The impact of the polyploidy in the essential oil traits can be related with the diversification and size of TPS gene family in *Psidium* species. The evolution of the TPS genes in the Myrtaceae family genomes have reported the largest TPS gene family in plants (*Eucalyptus* spp. having up to 100 genes)^26,39^ and occurrence of lineage-specific pathways and products. Although the essential oil of *Psidium species* exhibits a great diversity in its chemotypes conditioned to environmental and genetic variations^24,27,40,41^, the evolution of TPS genes in Myrtaceae neotropical fresh fruits remain unknown.

The increase of the values of 2C nuclear, CG% and 5-mC% was related to the decrease in (E)-Nerolidol and β-Bisabolol. Therefore, in addition to the genome effect (2n chromosome number, 2C nuclear value and GC%), chemical changes of the cytosine (5-mC) also influences composition of the essential oils. So, we showed the influence of the epigenetic control in the compound biosynthesis of the secondary metabolism in *Psidium*. The higher abundance of oxygenated sesquiterpenes was related to the occurrence of smaller genomes, with lower CG% and 5-mC%, indicating the genomic and epigenomic influence in this chemical class. In previous studies, the presence of oxygenated sesquiterpenes was clearly increased at the expense of hydrocarbons sesquiterpenes in spring in *P. guajava* genotypes^42^. Together, these data, which were reported for the first time, show the influence of genome and epigenome on essential oil yield and in specific compounds, suggesting for epigenetic control for terpene in Myrtaceae.

## Conclusion

From genome and epigenome to secondary metabolism, we provided data about the diversity of the *Psidium* species. We characterize the *Psidium* germplasm in relation to the 2n chromosome number, 2C nuclear and GC% values, TPS gene copy number and 5-mC%, generating knowledge about species previously studied and also about others not yet evaluated. In addition, we also explore the secondary metabolism, evidence the phenotypic divergences between *Psidium* species and individuals, and confirm our hypothesis about the influence of the genome and epigenome. Therefore, this work provides an important characterization of the genus *Psidium*, bringing information and evidence that can be incorporated in further studies, especially in phenotypic responses related to characters of economic interest.

## Material and methods

### Plant material

We collected leaf samples from ten *Psidium* species: *Psidium acidum* (DC.) Landrum, *P. cattleyanum* Sabine, *P. guajava* L., *P. guineense* Sw., *P. myrtoides* O.Berg*, P. gaudichaudianum* Proença & Faria*, P. friedrichsthalianum* (O.Berg) Nied*, P. macahense* O.Berg*, P. oblongatum* O.Berg, and *P. rufum* Mart. ex DC. Leaves were also collected from hybrids of *P. guajava x P. guineense*. Individuals not identified by species were kept and denominated as genus *Psidium* (Psi). Brazilian region of occurrence, phytogeographic domain and 2n chromosome number reported for the species are presented in Table 1. The number of individuals of each species for each analysis is presented in Supplementary Table S1. The localization of occurrence of each access, individual identification and families are presented in Supplementary Table S2.

### Nuclear 2C value and GC%

Young leaves from each germplasm (Supplementary Table S2) were used for nuclear 2C value and GC% measurements. *Solanum lycopersicum* L., 1753, ‘Stupické’ was used as internal standard (2C = 2.00 pg)^10^. 2 cm^2^ leaf fragment from each *Psidium* germplasm and from the *S. lycopersicum* were simultaneously chopped^43^ for about 30 s in a Petri dish containing 0.5 mL OTTO-I ^44^ modified for species of the Myrtaceae family (0.1 M citric acid, 0.5% Tween 20, 50 µg mL^-1^ RNAse, 2 mM dithiothreitol, and 7% polyethylene glycol 2000 – PEG)^37^.After adding 0.5 mL of the same buffer, the resulted suspensions were incubated for 3 min, filtered on a 30 μm diameter nylon filter (Partec) in a 2.0 mL microtube, and centrifuged at 100 *x*g for 5 min. The supernatant was discarded and 100 μL of the same buffer was added to the pellet, which was homogenized in vortex and incubated for 10 min. Subsequently, 0.5 mL of modified OTTO-II staining buffer (400 mM Na_2_HPO_4_H_2_O, 2 mM dithiothreitol, 50 µg mL^-1^ RNAse, and 75 µg mL^-1^ propidium iodide (PI, excitation/emission wavelengths: 480–575/550–740 nm) was added to the^10,44^. The suspensions were filtered through 20 µm nylon mesh (Partec) into tubes (Partec) and kept for 30 min in the dark. Then, the suspensions were analyzed in a flow cytometer (BD Accuri C6 flow cytometer, Accuri cytometers, Belgium) equipped with a 488 nm laser source to promote emissions at FL2 (615—670 nm) and FL3 (> 670 nm). The fluorescence peaks of the G_0_/G_1_ nuclei of each access and the standard were identified in the histograms using BD Accuri™ C6 software. G_0_/G_1_ peaks with coefficient of variation (CV) less than 5% were considered for nuclear 2C value measurement in pg by the formula: nuclear 2C value of the access (pg) = [(mean G_0_/G_1_ peak channel of the access)*2.00 pg S. *lycopersicum*]/(mean G_0_/G_1_ peak channel of *S. lycopersicum*).

For GC%, nuclear suspensions were generated following the procedure adopted to measure the nuclear 2C value with some modifications: (a) the OTTO I and II buffers were not supplemented with RNAse, and (b) the OTTO II buffer was supplemented with 1.5 μM of 4’,6-diamidino-2-phenylindole (DAPI, excitation/emission wavelengths: 320–385/400–580 nm). The suspensions were analyzed with a Partec PAS flow cytometer (Partec GmbH, Munster, Germany), equipped with an 388 nm UV mercury arc lamp and a GG 435–500 nm band-pass filter. AT% was measured using the formula^45^%AT_sample_ = %AT_standard_*[(R_DAPI_/R_PI_)^1/r^], in which: %AT_*S. lycopersicum*_ = 64.50% ^11,46^; R = ratio of the fluorescence intensity of the access/standard; r = 3 for DAPI^46^. From the AT%, the GC% was calculated by the following formula: GC% = 100—AT%. The data corresponding to the nuclear 2C value and GC% of *Psidium* accesses were submitted to clustering by the Toucher method optimized by Euclidean distance, in which the variables were separately evaluated (nuclear 2C value and GC%). The analyses were conducted in the Genes computer program^45^.

### Percentage of methylated cytosines (5-mC%) in the genome

The 5-mC% data of *P. guajava* accesses were revisited from our previous study^47^. For *P. cattleyanum*, *P. guineense*, *P. myrtoides*, *P. gaudichaudianum*, *P. friedrichsthalianum* and *Psidium* sp. the unpublished 5-mC% was measured based on the methodology used for *P. guajava*^47^.

### Yield and chemical composition of the essential oil

We revisited the data about yield and chemical composition of the essential oil previously published by our research group for *P. guajava*^40,42^, *P. guineense*^48^ and *P. cattleyanum*^24^. For the other accesses, the essential oil was extracted based on the methodology used for *P. guajava*^41^. The identification and semi-quantification of the leaf essential oil compounds were performed using gas chromatography with flame ionization detector (GC-FID QP2010SE, Shimadzu, Japan) and gas chromatography coupled to mass spectrometry (GC–MS QP2010SE, Shimadzu, Japan). For these analyses, the following conditions were adopted: the carrier gas used was He for both detectors with flow rate and linear velocity of 2.80 mL min^− 1^ and 50.80 cm sec^− 1^ (GC-FID) and 1.98 mL min^− 1^ and 50.90 cm sec^− 1^ (GC–MS), respectively; injector temperature was 220 °C at a split ratio of 1: 30; fused silica capillary column (30 m × 0.25 mm); Rtx-5MS stationary phase (0.25 μm film thickness); the oven temperature had the following programming: initial temperature of 40 °C, which remained for 3 min and then the temperature was gradually increased at 3 °C min^− 1^ until it reached 180 °C, remaining for ten minutes, with a total analysis time of 59.67 min; the temperatures used in the FID and MS detectors are 240 and 200 °C, respectively. The sample used was drawn from the vial in a volume of 1 μL of a 3% solution of essential oil dissolved in 95% hexane.

GC–MS analyses were performed in an electron impact equipment with an energy of 70 eV; scanning speed of 1000; scanning interval of 0.50 fragments.sec^− 1^ and detected fragments from 29 to 400 (*m/z*). GC-FID analyses were performed by a flame formed by H_2_ and atmospheric air with a temperature of 300 °C. Flow rates of 40 mL min^− 1^ and 400 mL min^− 1^ were used for H_2_ and air, respectively.

Identification of the essential oil compounds was performed by comparing the mass spectra in relation to available in the spectrophotometer database (Wiley 7, NIST 05 and NIST 05 s) and by the retention index (RI). For the RI calculation, a mixture of saturated C7-C40 alkanes (Supelco, USA) submitted under the same chromatographic conditions as the OE was used and the adjusted retention time of each compound was obtained using GC-FID. Then, the calculated values for each compound were compared with those in the literature^49–51^.

### Correlation analysis

2C value, GC%, 5-mC%, yield and content of each compound present in the essential oil were subjected to Pearson’s correlation. The analysis was conducted in the R environment^39^ using the package “Agricolae” (https://CRAN.R-project.org/package=agricolae).

### Terpene synthase gene (TPS) copy number in P. guajava and P. guineense

We showed the polyploidy influence in the copy number of the genes involved with essential oil synthesis, the terpene synthase genes (TPS). These genes encode enzymes that act in essential oil synthesis pathways^27,39,52–54^. For this, we used the sequence of genes functionally characterized and involved in the synthesis of terpenes (TPS genes), which have been described and available in database ID AB266390.1 and ID MK873024.1. Through the BLAST tool, the similarity of these sequences was evaluated in relation to the TPS genes from the *P. guajava* genome annotation (data of the research group). The alignments that presented a score of at least 80% were selected for the design of the primers. From this, the primers were designed in the conserved motives of these TPS genes, considering mainly exon regions*.* Primers were designed and evaluated using the OligoIDTAnalyzer program (IDT). We defined two pairs of primers: the first (*F* 5’-GGTGGGATGTCGATGCTAAA-3’ and *R* 5’-CTCTTCCTCCGTAACTCTGTATTG3’) specific to one predicted TPS gene orthologue with an amplicon 500 pb; and a general primer pair (*F* 5’-CGATTCCGGCTACTTAGACATC-3’ and *R* 5’-GTTCTTCCAGCGTCCCATATAC-3’) aligned to the conserved motifs in eight predicted TPS genes of *P. guajava* genome, corresponding to sequences from 415 to 502 pb.

The DNA sequences of the putative TPS were amplified from *P. guajava* and *P. guineense* genomic DNA using the primers. Amplification reaction consisted of 50 ng genomic DNA, 200 µM dNTPs, 0.5 µM each *R* and *F* primers, 1 U GoTaq enzyme (Promega), 1X GoTaq enzyme reaction buffer and 1.8 mM MgCl_2_. Amplification conditions were initial denaturation at 95 °C for 5 min, followed by 30 cycles of 95 °C for 1 min, 58 °C for 45 s, 72 °C for 1 min and a final extension at 72 °C for 5 min. The amplification products were evaluated on 1.5% agarose gel and NanoDrop. Then, DNA probes were generated for each putative gene by a second PCR reaction on the same conditions described above, differing by the labeling with Tetramethyl-rhodamine 5-dUTP (Roche) for the specific or ChromaTide Alexa Fluor 488–5-dUTP (Life Technologies) for the general. Fluorescent in situ hybridization (FISH) was performed in slides containing isolated and preserved nuclei to detect the number of hybridization signals corresponding to the TPS genes. Hybridization mix consisted of 50% formamide, 2X SSC and 200 ng of the probe. This mix was applied to the slide, which was covered with a coverslip, sealed with rubber cement and kept at 37 °C for 20 h. Post-hybridization wash was in 2X SSC at 42 °C for 20 min. Slides were counterstained with 4′,6-diamidino-2-phenylindole and analyzed on a photomicroscope Olympus BX60 equipped with epifluorescence and an immersion objective 100x/A.N. 1.4. At least 20 nuclei were scrambled for each species and for each gene using a 12-bit CCD digital video camera (Olympus) coupled to the photomicroscope and a computer with a digitizer plate. Captured images were processed by Image ProPlus 6.1 (Media Cybernetics).

### Ethical approval

This article does not contain any studies with human participants or animals performed by any of the authors.

## Supplementary Information

Supplementary Information.

## Data Availability

The datasets generated during and/or analysed during the current study are available from the corresponding author on reasonable request.
