# Supplementary material for: Genomic and epigenomic variation in Psidium species and their outcome under the yield and composition of essential oils
Source: Sci Rep. 2023 Jan 25;13:1385. doi: 10.1038/s41598-023-27912-w (PMC9876884; doi:10.1038/s41598-023-27912-w)
Supplement: Supplementary file 1 — Supplementary Information. [file 41598_2023_27912_MOESM1_ESM.pdf]

ORIGINAL ARTICLE

Genomic and epigenomic variation in *Psidium* species and their outcome under the yield and composition of essential oils.

Matheus Alves Silva[1], Fernanda Aparecida Ferrari Soares[2], Wellington Ronildo Clarindo[2], Luiza Alves Mendes[3], Luziane Brandão Alves[1], Adésio Ferreira[1], Marcia Flores da Silva Ferreira[*1]

[1]Universidade Federal do Espírito Santo – Centro de Ciências Agrárias e Engenharias – Departamento de Agronomia – Alto Universitário, s/n, Guararema – Alegre-ES, ZIP CODE: 29500-000 - Brasil

[2]Universidade Federal de Viçosa – Departamento de Biologia Geral – Av. Peter Henry Rolfs, s/n, Campus Universitário – Viçosa-MG, ZIP CODE: 36570-900 - Brasil

[3]Universidade Federal de Viçosa – Departamento de Química – Av. Peter Henry Rolfs, s/n, Campus Universitário – Viçosa-MG, ZIP CODE: 36570-900 - Brasil

[*]Corresponding author: marcia.ferreira@ufes.br

Tel.: +55 28 3552 86788

Supplementary Table S1. Number of *Psidium* accesses used for measuring nuclear DNA content (2C value), percentage of GC bases (GC%), percentage of methylated cytosines (5-mC%), yield (Yield), and essential oil composition (Comp).

Species	Identification of the half-sib family	Number of accesses							
		Total	Total		Half-sib		5-mC%	Yield	Comp
			2C value	CG %	2C value	CG %			
P. acidum (DC.) Landrum	-	4	4	2	-	-	-	-	-
P. cattleyanum Sabine	01	104	104	35	12	-	6	6	6
	02				5	-			
	03				30	5			
	04				30	2			
P. guajava L.	05	54	54	51	6	6	21	21	21
	06				17	17			
P. guajava L. x *P. guineense* Sw.	-	15	15	4	-	-	-	-	-
P. guineense Sw.	-	7	7	5	-	-	2	-	-
P. myrtoides O.Berg	07	54	54	26	18	18	5	5	5
	08				30	2			
P. gaudichaudianum Proença & Faria	09	30	30	6	29	5	1	1	1
P. friedrichsthalianum (O.Berg) Nied	-	1	1	1	-	-	1	1	1
P. macahense O.Berg	-	1	1	1	-	-	-	-	-
P. oblongatum O.Berg	-	1	1	1	-	-	-	-	-
P. rufum Mart. ex DC.	-	1	1	-	-	-	-	-	-
Psidium sp.	-	11	11	5	-	-	1	1	1
Total of accesses	-	283	283	137	177	55	37	35	35

Supplementary Table S2. Location, individual and half-sib family identification, nuclear DNA content (2C value) and percentage of GC bases (GC%) of *Psidium* accesses.

Species	Municipality of collection	Access	Family of half-sibs	2C value (pg)	CG%
P. acidum	Alegre - ES	Aci_01	-	2.07	40.54
P. acidum	Alegre - ES	Aci_02	-	2.08	40.04
P. acidum	Viçosa - MG	Aci_03	-	2.00	-
P. acidum	Viçosa - MG	Aci_04	-	2.00	-
P. cattleyanum	Alegre - ES	Cat_01	-	4.47	47.71
P. cattleyanum	Alegre - ES	Cat_02	-	4.74	48.16
P. cattleyanum	Alegre - ES	Cat_03	-	4.74	48.91
P. cattleyanum	Alegre - ES	Cat_04	-	3.77	40.57
P. cattleyanum	Alegre - ES	Cat_05	-	3.39	37.50
P. cattleyanum	Alegre - ES	Cat_06	-	3.51	39.62
P. cattleyanum	Alegre - ES	Cat_07	-	3.28	37.20
P. cattleyanum	Alegre - ES	Cat_08	-	3.43	38.17
P. cattleyanum	Alegre - ES	Cat_09	-	3.42	38.81
P. cattleyanum	Alegre - ES	Cat_10	-	3.43	38.96
P. cattleyanum	Alegre - ES	Cat_11	-	3.47	39.42
P. cattleyanum	Alegre - ES	Cat_12	-	3.44	38.64
P. cattleyanum	Alegre - ES	Cat_13	-	3.57	39.91
P. cattleyanum	Alegre - ES	Cat_14	-	3.47	39.50

P. cattleyanum	Alegre - ES	Cat_15	-	3.59	39.49
P. cattleyanum	Alegre - ES	Cat_16	-	3.74	40.71
P. cattleyanum	Alegre - ES	Cat_17	-	2.00	36.37
P. cattleyanum	Alegre - ES	Cat_18	-	3.44	39.12
P. cattleyanum	Alegre - ES	Cat_19	-	4.98	39.60
P. cattleyanum	Alegre - ES	Cat_20	-	6.83	42.45
P. cattleyanum	Alegre - ES	Cat_21	-	7.03	41.78
P. cattleyanum	Alegre - ES	Cat_22	-	4.26	40.98
P. cattleyanum	Alegre - ES	Cat_23	-	3.66	40.31
P. cattleyanum	Alegre - ES	Cat_24	-	4.71	38.70
P. cattleyanum	Alegre - ES	Cat_25	-	3.31	38.50
P. cattleyanum	Alegre - ES	Cat_26	-	3.45	39.05
P. cattleyanum	Viçosa - MG	Cat_27	01	3.62	-
P. cattleyanum	Viçosa - MG	Cat_28	01	4.21	-
P. cattleyanum	Viçosa - MG	Cat_29	01	4.22	-
P. cattleyanum	Viçosa - MG	Cat_30	01	4.22	-
P. cattleyanum	Viçosa - MG	Cat_31	01	3.60	-
P. cattleyanum	Viçosa - MG	Cat_32	01	3.57	-
P. cattleyanum	Viçosa - MG	Cat_33	01	3.44	-
P. cattleyanum	Viçosa - MG	Cat_34	01	5.68	-
P. cattleyanum	Viçosa - MG	Cat_35	01	3.41	-

P. cattleyanum	Viçosa - MG	Cat_36	01	3.29	-
P. cattleyanum	Viçosa - MG	Cat_37	01	3.64	-
P. cattleyanum	Viçosa - MG	Cat_38	01	3.45	-
P. cattleyanum	Alegre - ES	Cat_39	-	3.701	40.47
P. cattleyanum	Viçosa - MG	Cat_40	02	3.85	-
P. cattleyanum	Viçosa - MG	Cat_41	02	3.98	-
P. cattleyanum	Viçosa - MG	Cat_42	02	3.45	-
P. cattleyanum	Viçosa - MG	Cat_43	02	3.97	-
P. cattleyanum	Viçosa - MG	Cat_44	02	3.82	-
P. cattleyanum	Alegre - ES	Cat_45	03	3.85	41.25
P. cattleyanum	Alegre - ES	Cat_46	03	3.63	-
P. cattleyanum	Alegre - ES	Cat_47	03	3.86	-
P. cattleyanum	Alegre - ES	Cat_48	03	3.95	41.46
P. cattleyanum	Alegre - ES	Cat_49	03	3.66	-
P. cattleyanum	Alegre - ES	Cat_50	03	3.68	-
P. cattleyanum	Alegre - ES	Cat_51	03	3.67	-
P. cattleyanum	Alegre - ES	Cat_52	03	3.39	-
P. cattleyanum	Alegre - ES	Cat_53	03	3.68	-
P. cattleyanum	Alegre - ES	Cat_54	03	3.68	-
P. cattleyanum	Alegre - ES	Cat_55	03	3.65	-
P. cattleyanum	Alegre - ES	Cat_56	03	3.39	-

P. cattleyanum	Alegre - ES	Cat_57	03	3.64	-
P. cattleyanum	Alegre - ES	Cat_58	03	3.60	39.87
P. cattleyanum	Alegre - ES	Cat_59	03	3.34	37.99
P. cattleyanum	Alegre - ES	Cat_60	03	3.52	39.21
P. cattleyanum	Alegre - ES	Cat_61	03	3.44	-
P. cattleyanum	Alegre - ES	Cat_62	03	3.66	-
P. cattleyanum	Alegre - ES	Cat_63	03	3.38	-
P. cattleyanum	Alegre - ES	Cat_64	03	3.68	-
P. cattleyanum	Alegre - ES	Cat_65	03	3.69	-
P. cattleyanum	Alegre - ES	Cat_66	03	3.64	-
P. cattleyanum	Alegre - ES	Cat_67	03	3.91	-
P. cattleyanum	Alegre - ES	Cat_68	03	3.65	-
P. cattleyanum	Alegre - ES	Cat_69	03	3.91	-
P. cattleyanum	Alegre - ES	Cat_70	03	3.59	-
P. cattleyanum	Alegre - ES	Cat_71	03	4.27	-
P. cattleyanum	Alegre - ES	Cat_72	03	3.54	39.89
P. cattleyanum	Alegre - ES	Cat_73	03	3.74	-
P. cattleyanum	Alegre - ES	Cat_74	03	3.86	-
P. cattleyanum	Alegre - ES	Cat_75	04	4.10	-
P. cattleyanum	Alegre - ES	Cat_76	04	4.31	-
P. cattleyanum	Alegre - ES	Cat_77	04	4.35	-

P. cattleyanum	Alegre - ES	Cat_78	04	4.06	-
P. cattleyanum	Alegre - ES	Cat_79	04	4.38	41.23
P. cattleyanum	Alegre - ES	Cat_80	04	4.34	-
P. cattleyanum	Alegre - ES	Cat_81	04	4.34	-
P. cattleyanum	Alegre - ES	Cat_82	04	4.05	-
P. cattleyanum	Alegre - ES	Cat_83	04	4.24	-
P. cattleyanum	Alegre - ES	Cat_84	04	4.28	-
P. cattleyanum	Alegre - ES	Cat_85	04	4.23	-
P. cattleyanum	Alegre - ES	Cat_86	04	4.31	-
P. cattleyanum	Alegre - ES	Cat_87	04	4.30	-
P. cattleyanum	Alegre - ES	Cat_88	04	4.27	-
P. cattleyanum	Alegre - ES	Cat_89	04	4.27	-
P. cattleyanum	Alegre - ES	Cat_90	04	4.26	-
P. cattleyanum	Alegre - ES	Cat_91	04	4.27	42.59
P. cattleyanum	Alegre - ES	Cat_92	04	4.25	-
P. cattleyanum	Alegre - ES	Cat_93	04	4.08	-
P. cattleyanum	Alegre - ES	Cat_94	04	4.08	-
P. cattleyanum	Alegre - ES	Cat_95	04	4.05	-
P. cattleyanum	Alegre - ES	Cat_96	04	3.63	-
P. cattleyanum	Alegre - ES	Cat_97	04	3.97	-
P. cattleyanum	Alegre - ES	Cat_98	04	4.11	-

P. cattleyanum	Alegre - ES	Cat_99	04	4.12	-
P. cattleyanum	Alegre - ES	Cat_100	04	4.05	-
P. cattleyanum	Alegre - ES	Cat_101	04	4.05	-
P. cattleyanum	Alegre - ES	Cat_102	04	4.03	-
P. cattleyanum	Alegre - ES	Cat_103	04	3.96	-
P. cattleyanum	Alegre - ES	Cat_104	04	4.08	-
P. guajava	Alegre - ES	Gua_01	05	0.98	37.81
P. guajava	Alegre - ES	Gua_02	05	0.99	39.58
P. guajava	Alegre - ES	Gua_03	05	0.95	38.09
P. guajava	Alegre - ES	Gua_04	05	0.96	37.32
P. guajava	Alegre - ES	Gua_05	05	0.95	38.44
P. guajava	Alegre - ES	Gua_06	05	1.03	40.33
P. guajava	Alegre - ES	Gua_07	06	0.98	39.29
P. guajava	Alegre - ES	Gua_08	06	0.95	38.60
P. guajava	Alegre - ES	Gua_09	06	0.95	37.64
P. guajava	Alegre - ES	Gua_10	06	0.96	38.29
P. guajava	Alegre - ES	Gua_11	06	0.98	37.71
P. guajava	Alegre - ES	Gua_12	06	0.96	38.93
P. guajava	Alegre - ES	Gua_13	06	0.92	36.24
P. guajava	Alegre - ES	Gua_14	06	1.00	38.95
P. guajava	Alegre - ES	Gua_15	06	0.99	38.47

P. guajava	Alegre - ES	Gua_16	06	0.94	38.39
P. guajava	Alegre - ES	Gua_17	06	0.95	37.66
P. guajava	Alegre - ES	Gua_18	06	0.94	36.24
P. guajava	Alegre - ES	Gua_19	06	0.95	37.42
P. guajava	Alegre - ES	Gua_20	06	0.95	36.91
P. guajava	Alegre - ES	Gua_21	06	0.96	38.60
P. guajava	Alegre - ES	Gua_22	06	0.99	37.45
P. guajava	Alegre - ES	Gua_23	06	0.99	37.76
P. guajava	Alegre - ES	Gua_24	-	0.95	38.73
P. guajava	Alegre - ES	Gua_25	-	0.99	40.00
P. guajava	Alegre - ES	Gua_26	-	0.95	38.09
P. guajava	Alegre - ES	Gua_27	-	0.95	38.69
P. guajava	Alegre - ES	Gua_28	-	0.95	38.80
P. guajava	Alegre - ES	Gua_29	-	0.95	38.38
P. guajava	Alegre - ES	Gua_30	-	0.95	36.72
P. guajava	Alegre - ES	Gua_31	-	0.95	36.72
P. guajava	Alegre - ES	Gua_32	-	0.95	36.95
P. guajava	Alegre - ES	Gua_33	-	0.95	38.69
P. guajava	Alegre - ES	Gua_34	-	0.95	37.57
P. guajava	Alegre - ES	Gua_35	-	0.94	37.96
P. guajava	Alegre - ES	Gua_36	-	0.95	36.85

P. guajava	Alegre - ES	Gua_37	-	0.95	37.34
P. guajava	Alegre - ES	Gua_38	-	0.95	36.95
P. guajava	Alegre - ES	Gua_39	-	0.95	37.88
P. guajava	Alegre - ES	Gua_40	-	0.95	36.85
P. guajava	Alegre - ES	Gua_41	-	0.95	36.16
P. guajava	Alegre - ES	Gua_42	-	0.95	37.63
P. guajava	Alegre - ES	Gua_43	-	0.95	37.68
P. guajava	Alegre - ES	Gua_44	-	0.95	36.95
P. guajava	Alegre - ES	Gua_45	-	0.95	37.34
P. guajava	Alegre - ES	Gua_46	-	0.95	36.85
P. guajava	Alegre - ES	Gua_47	-	1.10	40.51
P. guajava	Alegre - ES	Gua_48	-	0.94	37.20
P. guajava	Alegre - ES	Gua_49	-	0.94	36.98
P. guajava	Alegre - ES	Gua_50	-	0.99	38.97
P. guajava	Alegre - ES	Gua_51	-	0.93	-
P. guajava	Alegre - ES	Gua_52	-	0.90	-
P. guajava	Alegre - ES	Gua_53	-	0.91	-
P. guajava	Alegre - ES	Gua_54	-	0.95	40.74
P. guajava x P. guineense	Alegre - ES	Hib_01	-	1.90	36.05
P. guajava x P. guineense	Alegre - ES	Hib_02	-	1.90	35.07
P. guajava x P. guineense	Alegre - ES	Hib_03	-	1.90	36.08

P. guajava x P. guineense	Alegre - ES	Hib_04	-	1.90	35.35
P. guajava x P. guineense	Alegre - ES	Hib_05	-	1.90	-
P. guajava x P. guineense	Alegre - ES	Hib_06	-	1.90	-
P. guajava x P. guineense	Alegre - ES	Hib_07	-	1.90	-
P. guajava x P. guineense	Alegre - ES	Hib_08	-	1.90	-
P. guajava x P. guineense	Alegre - ES	Hib_09	-	1.90	-
P. guajava x P. guineense	Alegre - ES	Hib_10	-	1.90	-
P. guajava x P. guineense	Alegre - ES	Hib_11	-	0.95 (30%)/1.90 (70%)	-
P. guajava x P. guineense	Alegre - ES	Hib_12	-	1.90	-
P. guajava x P. guineense	Alegre - ES	Hib_13	-	1.90	-
P. guajava x P. guineense	Alegre - ES	Hib_14	-	1.90	-
P. guajava x P. guineense	Alegre - ES	Hib_15	-	1.90	-
P. guineense	Alegre - ES	Gui_01	-	1.80	34.33
P. guineense	Alegre - ES	Gui_02	-	1.90	35.37
P. guineense	Alegre - ES	Gui_03	-	1.90	35.36
P. guineense	Alegre - ES	Gui_04	-	2.00	36.14
P. guineense	Alegre - ES	Gui_05	-	2.00	43.07
P. guineense	Viçosa - MG	Gui_06	-	1.84	-
P. guineense	Viçosa - MG	Gui_07	-	1.85	-
P. myrtoides	Alegre - ES	Myr_01	-	2.97	38.63
P. myrtoides	Alegre - ES	Myr_02	-	2.96	39.52

P. myrtoides	Alegre - ES	Myr_03	-	2.87	39.94
P. myrtoides	Alegre - ES	Myr_04	-	2.87	37.63
P. myrtoides	Alegre - ES	Myr_05	-	2.88	38.44
P. myrtoides	Alegre - ES	Myr_06	-	3.02	39.34
P. myrtoides	Alegre - ES	Myr_07	07	2.98	38.72
P. myrtoides	Alegre - ES	Myr_08	07	3.02	39.54
P. myrtoides	Alegre - ES	Myr_09	07	3.02	40.12
P. myrtoides	Alegre - ES	Myr_10	07	3.02	39.23
P. myrtoides	Alegre - ES	Myr_11	07	3.01	39.40
P. myrtoides	Alegre - ES	Myr_12	07	2.93	38.37
P. myrtoides	Alegre - ES	Myr_13	07	2.94	38.66
P. myrtoides	Alegre - ES	Myr_14	07	3.03	39.35
P. myrtoides	Alegre - ES	Myr_15	07	2.82	38.59
P. myrtoides	Alegre - ES	Myr_16	07	3.02	39.10
P. myrtoides	Alegre - ES	Myr_17	07	3.02	39.72
P. myrtoides	Alegre - ES	Myr_18	07	3.02	40.49
P. myrtoides	Alegre - ES	Myr_19	07	2.95	39.01
P. myrtoides	Alegre - ES	Myr_20	07	3.01	39.05
P. myrtoides	Alegre - ES	Myr_21	07	3.02	39.70
P. myrtoides	Alegre - ES	Myr_22	07	3.05	39.67
P. myrtoides	Alegre - ES	Myr_23	07	3.03	40.24

P. myrtoides	Alegre - ES	Myr_24	07	3.09	39.92
P. myrtoides	Alegre - ES	Myr_25	08	3.05	-
P. myrtoides	Alegre - ES	Myr_26	08	3.12	48.95
P. myrtoides	Alegre - ES	Myr_27	08	2.96	-
P. myrtoides	Alegre - ES	Myr_28	08	2.98	-
P. myrtoides	Alegre - ES	Myr_29	08	2.88	-
P. myrtoides	Alegre - ES	Myr_30	08	2.98	-
P. myrtoides	Alegre - ES	Myr_31	08	3.01	-
P. myrtoides	Alegre - ES	Myr_32	08	2.96	-
P. myrtoides	Alegre - ES	Myr_33	08	2.95	-
P. myrtoides	Alegre - ES	Myr_34	08	3.07	-
P. myrtoides	Alegre - ES	Myr_35	08	3.01	-
P. myrtoides	Alegre - ES	Myr_36	08	2.96	-
P. myrtoides	Alegre - ES	Myr_37	08	3.02	-
P. myrtoides	Alegre - ES	Myr_38	08	2.98	-
P. myrtoides	Alegre - ES	Myr_39	08	2.97	-
P. myrtoides	Alegre - ES	Myr_40	08	2.72	-
P. myrtoides	Alegre - ES	Myr_41	08	2.76	-
P. myrtoides	Alegre - ES	Myr_42	08	2.76	-
P. myrtoides	Alegre - ES	Myr_43	08	2.91	-
P. myrtoides	Alegre - ES	Myr_44	08	2.77	-

P. myrtoides	Alegre - ES	Myr_45	08	2.96	-
P. myrtoides	Alegre - ES	Myr_46	08	2.91	-
P. myrtoides	Alegre - ES	Myr_47	08	2.95	-
P. myrtoides	Alegre - ES	Myr_48	08	2.93	-
P. myrtoides	Alegre - ES	Myr_49	08	2.99	-
P. myrtoides	Alegre - ES	Myr_50	08	2.85	-
P. myrtoides	Alegre - ES	Myr_51	08	2.83	-
P. myrtoides	Alegre - ES	Myr_52	08	2.92	-
P. myrtoides	Alegre - ES	Myr_53	08	3.02	-
P. myrtoides	Alegre - ES	Myr_54	08	2.77	38.71
P. gaudichaudianum	Alegre - ES	Gau_01	-	4.91	39.47
P. gaudichaudianum	Alegre - ES	Gau_02	09	4.91	-
P. gaudichaudianum	Alegre - ES	Gau_03	09	4.93	-
P. gaudichaudianum	Alegre - ES	Gau_04	09	4.85	-
P. gaudichaudianum	Alegre - ES	Gau_05	09	4.83	-
P. gaudichaudianum	Alegre - ES	Gau_06	09	4.86	-
P. gaudichaudianum	Alegre - ES	Gau_07	09	4.64	38.38
P. gaudichaudianum	Alegre - ES	Gau_08	09	4.84	-
P. gaudichaudianum	Alegre - ES	Gau_09	09	5.15	40.48
P. gaudichaudianum	Alegre - ES	Gau_10	09	4.83	-
P. gaudichaudianum	Alegre - ES	Gau_11	09	4.81	-

P. gaudichaudianum	Alegre - ES	Gau_12	09	4.90	-
P. gaudichaudianum	Alegre - ES	Gau_13	09	5.02	-
P. gaudichaudianum	Alegre - ES	Gau_14	09	4.86	-
P. gaudichaudianum	Alegre - ES	Gau_15	09	5.14	-
P. gaudichaudianum	Alegre - ES	Gau_16	09	4.83	-
P. gaudichaudianum	Alegre - ES	Gau_17	09	4.79	-
P. gaudichaudianum	Alegre - ES	Gau_18	09	5.02	39.97
P. gaudichaudianum	Alegre - ES	Gau_19	09	4.81	-
P. gaudichaudianum	Alegre - ES	Gau_20	09	5.04	-
P. gaudichaudianum	Alegre - ES	Gau_21	09	4.73	40.06
P. gaudichaudianum	Alegre - ES	Gau_22	09	4.76	-
P. gaudichaudianum	Alegre - ES	Gau_23	09	4.79	-
P. gaudichaudianum	Alegre - ES	Gau_24	09	5.19	-
P. gaudichaudianum	Alegre - ES	Gau_25	09	4.87	-
P. gaudichaudianum	Alegre - ES	Gau_26	09	5.12	-
P. gaudichaudianum	Alegre - ES	Gau_27	09	4.79	-
P. gaudichaudianum	Alegre - ES	Gau_28	09	4.83	-
P. gaudichaudianum	Alegre - ES	Gau_29	09	7.40	39.68
P. gaudichaudianum	Alegre - ES	Gau_30	09	5.17	-
P. friedrichsthalianum	Alegre - ES	Fri_01	-	4.72	38.59
P. macahense	Alegre - ES	Mac_01	-	0.93	37.56

P. oblongatum	Alegre - ES	Obl_01	-	0.99	39.81
P. rufum	Viçosa - MG	Ruf_01	-	4.23	-
Psidium sp.	Alegre - ES	Psi_01	-	4.94	40.92
Psidium sp.	Alegre - ES	Psi_02	-	4.22	37.22
Psidium sp.	Alegre - ES	Psi_03	-	4.92	38.80
Psidium sp.	Alegre - ES	Psi_04	-	2.00	-
Psidium sp.	Alegre - ES	Psi_05	-	2.00	-
Psidium sp.	Alegre - ES	Psi_06	-	2.00	-
Psidium sp.	Alegre - ES	Psi_07	-	2.00	36.80
Psidium sp.	Alegre - ES	Psi_08	-	2.00	-
Psidium sp.	Alegre - ES	Psi_09	-	2.00	-
Psidium sp.	Alegre - ES	Psi_10	-	1.92	35.90
Psidium sp.	Viçosa - MG	Psi_11	-	4.84	-

Supplementary Table S3. Percentage of methylated cytosines (%5-mC), yield (Yield) and compounds identified in *Psidium* essential oil.

Genotype identification /Variable	Cat_20	Cat_21	Cat_22	Cat_25	Cat_26	Cat_39	Gua_26	Gua_27	Gua_28	Gua_29	Gua_30	Gua_31	Gua_32	Gua_33	Gua_34	Gua_35	Gua_36	Gua_37	Gua_38	Gua_39	Gua_40	Gua_41	Gua_42	Gua_44	Gua_45	Gua_46	Gua_54	Gui_02	Gui_03	Myr_01	Myr_02	Myr_03	Myr_04	Myr_05	Gau_01	Fri_01	Psi_01
%5-mC	24.88	17.02	23.80	26.17	30.00	26.67	19.45	21.03	18.35	19.65	19.91	16.70	19.74	20.00	21.34	17.01	20.63	21.47	19.79	19.73	16.34	19.79	20.44	22.26	26.19	21.12	22.30	19.39	21.21	22.39	33.30	26.57	24.96	17.77	21.95	23.94	22.62
Yield	0.95	0.90	0.75	0.70	0.73	0.70	0.54	0.40	0.22	0.52	0.32	0.20	0.34	0.42	0.30	0.44	0.42	0.47	0.35	0.30	0.54	0.44	0.41	0.39	0.42	0.32	0.51	0.28	0.3	0.31	0.40	0.40	0.38	0.37	0.52	0.57	0.47
Limonene[1]	-	-	-	-	-	2.05	0.70	0.10	5.88	0.25	12.12	7.65	0.30	-	-	7.45	6.48	5.53	11.43	-	0.20	3.22	8.85	1.20	0.50	0.25	6.55	*	*	-	-	-	-	-	18.30	-	-
α-Pinene[1]	6.06	2.03	10.35	23.80	19.56	5.7	-	-	-	-	-	-	-	-	-	-	-	-	-	-	-	-	-	-	-	-	-	*	*	30.70	28.90	21.20	21.40	21.40	10.60	30.70	16.60
β-Pinene[1]	-	-	-	3.62	-	-	-	-	-	-	-	-	-	-	-	-	-	-	-	-	-	-	-	-	-	-	-	*	*	-	-	-	-	-	-	-	-
Myrcene[1]	-	-	8.16	-	-	2.01	-	-	-	-	-	-	-	-	-	-	-	-	-	-	-	-	-	-	-	-	-	*	*	-	-	-	-	-	-	-	-
β-ocimene[1]	-	-	-	5.20	4.17	-	-	-	-	-	-	-	-	-	-	-	-	-	-	-	-	-	-	-	-	-	-	*	*	-	-	-	-	-	-	-	-
γ-Terpinene[1]	-	-	-	-	-	-	-	-	-	-	-	-	-	-	-	-	-	-	-	-	-	-	-	-	-	-	-	*	*	-	-	-	-	-	6.70	-	-
1,8-cineol[2]	-	-	-	27.7	24.34	-	4.45	1.93	2.52	3.63	3.36	3.46	3.35	1.23	2.56	3.34	2.98	2.32	3.07	1.85	3.23	2.48	2.50	3.45	4.12	3.96	2.10	*	*	-	-	-	-	-	16.30	31.10	6.90
α-Terpineol[2]	-	-	-	4.38	3.45	-	1.44	0.20	0.45	0.50	0.60	0.45	0.45	0.20	0.35	0.45	0.30	-	0.35	0.25	0.40	0.55	0.30	0.70	0.55	1.40	0.35	*	*	-	-	-	-	-	-	-	-
2-Methylbutyl 2-Methylbutanoate[2]	-	-	-	-	-	-	-	-	-	-	-	-	-	-	-	-	-	-	-	-	-	-	-	-	-	-	-	*	*	-	-	-	-	-	-	-	17.30
α-Copaene[3]	9.90	9.88	-	-	-	10.2	1.38	0.30	0.60	-	-	0.70	-	0.30	0.40	-	-	-	-	-	-	0.30	0.40	1.82	0.30	1.68	-	*	*	-	-	-	-	-	-	-	-
α-Cedrene[3]	-	-	-	-	-	-	-	1.25	-	-	0.60	-	0.65	1.05	1.15	0.55	0.90	0.70	0.85	0.90	-	1.05	1.00	-	-	-	0.65	*	*	-	-	-	-	-	-	-	-
cis-α-Bergamotene[3]	-	-	-	-	-	-	-	1.10	-	-	0.45	-	0.55	0.75	0.90	0.60	0.80	0.60	0.80	0.80	0.40	0.90	0.85	-	-	-	0.50	*	*	-	-	-	-	-	-	-	-
α-Humulene[3]	13.38	13.93	6.68	-	3.11	13.06	2.94	12.42	4.30	12.52	9.40	4.54	19.93	12.68	12.50	12.60	15.68	18.47	1.77	12.85	14.47	10.73	1.80	3.18	2.12	3.22	18.03	*	*	10.20	12.50	12.40	12.2	15.10	7.70	-	-
γ-Muurolene[3]	-	-	-	-	-	-	-	5.40	-	-	2.45	-	1.65	5.20	5.30	1.85	3.30	2.70	2.85	3.70	-	3.45	3.60	-	-	-	1.60	*	*	-	-	-	-	-	-	-	-
β-Selinene[3]	-	-	-	-	-	6.39	6.07	0.50	0.50	7.37	-	0.60	0.65	-	-	7.27	-	0.70	3.10	-	9.52	-	1.05	10.72	9.08	6.80	0.55	*	*	-	-	-	-	-	-	-	-
α-Selinene[3]	-	-	-	-	-	2.9	5.25	0.40	-	6.92	-	0.60	0.65	-	-	5.50	-	0.70	3.08	-	7.85	-	1.00	9.42	7.57	6.03	0.55	*	*	-	-	-	-	-	-	-	-
α-Bisabolene[3]	-	-	-	-	-	-	-	1.90	0.50	-	1.94	-	1.22	2.06	2.02	0.50	1.30	1.27	1.93	1.55	-	1.95	1.80	-	-	-	1.52	*	*	-	-	-	-	-	-	-	-
β-Bisabolene[3]	-	-	-	-	-	-	-	7.27	1.00	-	4.52	2.90	5.00	9.00	8.63	3.60	6.67	5.67	6.05	6.72	-	7.27	7.88	-	-	-	5.17	*	*	-	-	-	-	-	-	-	-
δ-Amorphene[3]	-	-	2.65	-	3.57	-	-	2.35	-	-	1.00	-	1.65	-	2.00	-	-	1.90	2.65	2.00	-	2.90	2.50	-	-	-	1.45	*	*	-	-	-	-	-	-	-	-
δ-Candinene[3]	6.34	6.78	-	-	-	-	1.05	4.85	0.50	-	1.35	0.50	1.90	3.70	3.75	0.90	2.55	1.95	3.00	2.55	-	3.40	3.65	1.50	0.95	1.25	1.60	*	*	-	-	-	-	-	-	-	-
(E)-α-Bisabolene[3]	-	-	-	-	-	-	-	2.55	0.50	-	0.95	-	1.45	1.00	1.80	0.85	2.00	1.45	2.00	1.75	-	2.30	2.30	-	-	-	1.30	*	*	-	-	-	-	-	-	-	-
Aromadendrene[3]	-	-	-	-	-	-	2.67	-	-	-	-	-	-	-	-	-	0.40	-	-	0.40	-	0.60	0.40	-	-	3.15	0.30	*	*	-	-	-	-	-	-	-	-
β-caryophyllene[3]	58.45	62.27	72.16	35.3	41.8	57.69	20.15	7.77	32.50	13.18	6.18	28.73	10.63	7.70	7.35	13.48	10.48	10.03	9.47	11.07	15.67	6.08	9.78	19.52	12.05	22.98	9.85	*	*	15.40	17.20	17.10	18.60	17.30	29.60	16.10	11.50
(E)-Farnesene[3]	-	-	-	-	-	-	-	1.30	-	-	1.80	-	-	1.85	1.80	-	-	-	-	-	-	1.60	2.40	-	-	-	-	*	*	-	-	-	-	-	-	-	-
α-Farnesene[3]	-	-	-	-	-	-	-	-	-	-	-	-	-	-	-	-	-	-	-	-	-	-	-	-	-	-	-	*	*	-	-	-	-	-	-	-	8.40
α-Acoradiene[3]	-	-	-	-	-	-	-	-	-	-	1.60	-	-	1.80	1.80	-	-	-	-	-	-	1.50	2.10	-	-	-	-	*	*	-	-	-	-	-	-	-	-
γ-Gurjunene[3]	-	-	-	-	-	-	-	-	-	-	-	-	-	-	1.40	-	-	-	-	-	-	-	-	1.15	-	-	-	*	*	-	-	-	-	-	-	-	-
γ-Curcumene[3]	-	-	-	-	-	-	-	1.50	-	-	1.30	-	-	1.60	-	-	-	-	-	-	-	1.40	-	-	-	-	-	*	*	-	-	-	-	-	-	-	-
α-Curcumene[3]	-	-	-	-	-	-	-	4.45	-	-	4.05	2.20	4.05	6.08	5.70	-	4.48	4.23	7.03	4.13	-	5.25	6.75	-	-	-	3.43	*	*	-	-	-	-	-	-	-	-
β-Himachalene[3]	-	-	-	-	-	-	-	2.80	-	-	4.30	-	1.85	3.43	3.27	-	2.10	1.95	2.57	2.13	-	2.63	2.17	-	-	-	2.03	*	*	-	-	-	-	-	-	-	-
β-Sesquiphellandrene[3]	-	-	-	-	-	-	-	3.50	-	-	3.43	-	2.05	4.88	4.50	-	2.63	2.85	3.40	3.05	-	3.35	4.97	1.63	-	-	2.55	*	*	-	-	-	-	-	-	-	-
γ-Candinene[3]	-	-	-	-	-	-	-	1.05	-	0.30	0.50	2.10	0.70	0.75	0.90	0.50	0.85	0.65	1.00	0.80	-	1.05	1.00	-	1.10	0.30	0.60	*	*	-	-	-	-	-	-	-	-
Elemol[4]	-	-	-	-	-	-	0.50	-	2.65	0.50	-	2.40	-	-	-	0.40	-	-	-	0.50	0.60	0.70	0.40	0.85	0.75	0.40	-	*	*	-	-	-	-	-	-	-	-
(E)-Nerolidol[4]	-	-	-	-	-	-	3.05	5.53	15.60	3.18	4.95	14.40	6.92	4.83	5.17	3.60	7.18	5.70	6.02	9.23	3.52	5.83	8.23	3.24	-	3.35	6.62	*	*	-	-	-	-	-	-	-	-
Caryophyllene oxide[4]	-	-	-	-	-	-	16.40	4.13	6.93	7.63	3.07	7.17	3.92	3.90	3.23	8.15	4.60	4.28	4.35	4.18	7.40	3.33	4.58	6.43	7.35	15.02	3.68	*	*	14.90	13.10	16.20	15.20	15.50	10.80	11.00	-
Ledol[4]	-	-	-	-	-	-	1.02	0.75	-	-	1.40	-	0.85	0.85	0.95	0.75	0.80	0.75	0.95	0.90	-	1.10	1.15	1.44	-	1.00	0.90	*	*	-	-	-	-	-	-	-	-
Humulene epoxide[4]	-	-	-	-	-	-	0.60	5.17	-	5.40	4.10	1.10	5.93	4.53	4.18	5.18	6.13	6.27	0.75	4.47	5.02	4.48	0.80	0.60	-	0.60	5.70	*	*	-	-	-	-	-	-	-	-
γ-Eudesmol[4]	-	-	-	-	-	-	3.53	3.30	4.28	9.16	2.33	3.95	3.90	2.38	2.38	8.28	3.94	4.28	1.33	2.72	7.34	2.67	1.43	3.27	3.87	3.18	3.68	*	*	-	-	-	-	-	-	-	-
epi-β-Cubenol[4]	-	-	-	-	-	-	3.18	3.60	-	1.70	2.63	3.40	2.43	3.25	3.25	1.45	2.50	2.58	3.03	2.73	1.70	3.13	3.33	3.50	6.55	3.18	2.50	*	*	-	-	-	-	-	-	-	-
epi-α-Cadinol[4]	-	-	-	-	-	-	2.10	2.23	4.00	8.90	2.90	4.00	1.10	2.47	2.27	6.90	4.10	5.17	1.78	3.13	7.57	3.18	1.95	2.63	5.58	1.98	5.85	*	*	-	-	-	-	-	-	-	-
Hinesol[4]	-	-	-	-	-	-	7.80	-	10.25	7.70	-	9.30	1.00	-	-	3.55	-	0.90	-	-	5.30	-	-	8.95	10.75	6.60	1.10	*	*	-	-	-	-	-	-	-	-
β-Eudesmol[4]	-	-	-	-	-	-	1.55	1.20	3.00	0.80	1.65	2.70	1.15	1.45	1.50	1.10	1.20	1.10	1.20	1.20	0.65	1.25	1.80	1.10	1.20	1.35	1.30	*	*	-	-	-	-	-	-	-	-
Selina-6-en-4-ol[4]	-	-	-	-	-	-	9.10	0.60	2.60	1.37	-	-	1.60	2.00	-	5.17	-	1.40	3.48	-	1.70	2.50	2.33	1.78	2.33	9.10	2.40	*	*	-	-	-	-	-	-	-	-
14-Hydroxy-epi-(E)-Caryophyllene[4]	-	-	-	-	-	-	9.30	2.40	3.20	13.85	4.35	2.65	2.45	2.80	2.85	-	2.55	2.50	1.70	2.75	11.95	3.10	3.10	14.40	17.00	8.20	1.05	*	*	-	-	-	-	-	-	-	-
β-Bisabolol[4]	-	-	-	-	-	-	2.97	19.17	3.90	2.32	18.63	3.72	14.27	18.13	18.07	4.23	14.18	13.92	18.15	17.98	2.02	19.37	18.62	2.48	3.20	2.55	13.95	*	*	-	-	-	-	-	-	-	-
α-Bisabolol[4]	-	-	-	-	-	-	0.50	2.88	1.25	1.15	5.87	1.45	3.20	3.56	3.32	4.00	4.06	3.45	3.40	2.95	1.05	3.27	3.07	1.05	1.05	0.50	3.83	*	*	15.50	16.10	18.20	19.30	22.00	-	-	-
trans-(2Z-6Z)-Farnesol[4]	-	-	-	-	-	-	-	0.70	3.20	-	0.85	3.50	0.65	0.90	1.25	-	0.65	0.55	0.50	0.55	-	0.70	0.90	-	-	-	0.55	*	*	-	-	-	-	-	-	-	-
α-Cedrol[4]	-	-	-	-	-	-	-	2.00	-	1.30	2.00	-	1.80	1.50	1.60	1.10	1.90	1.80	-	1.60	1.20	1.90	-	-	-	-	1.60	*	*	-	-	-	-	-	-	-	-
Aromadendrene epoxide[4]	-	-	-	-	-	-	11.20	3.80	10.80	8.48	2.60	12.03	2.60	2.70	2.40	7.90	2.53	-	2.75	2.70	-	2.80	3.05	8.38	12.33	10.25	2.20	*	*	7.10	6.40	7.50	6.70	8.70	-	6.40	7.20
α-Muurolol[4]	-	-	-	-	-	-	2.25	1.60	3.33	1.30	-	3.13	-	-	1.50	1.20	-	-	-	1.35	1.30	-	1.93	1.73	2.53	2.13	-	*	*	-	-	-	-	-	-	-	-
Selin-11-en-4a-ol[4]	-	-	-	-	-	-	8.75	1.80	3.30	11.73	-	3.55	1.60	-	-	9.85	1.80	1.75	4.60	-	10.60	-	2.47	15.10	20.15	8.15	1.30	*	*	-	-	-	-	-	-	-	-
(Z,E)-α-Bergamotol[4]	-	-	-	-	-	-	-	4.58	-	-	4.23	-	4.55	4.48	4.08	-	3.30	2.85	5.35	5.45	-	4.43	5.18	-	-	-	2.50	*	*	-	-	-	-	-	-	-	-
(2Z,6E)-Farnesol[4]	-	-	-	-	-	-	-	1.20	2.15	-	-	2.20	-	-	1.20	-	-	-	-	-	-	-	-	-	-	-	-	*	*	-	-	-	-	-	-	-	-
Viridiflorol[4]	-	2.61	-	-	-	-	-	-	-	-	-	-	-	-	-	-	-	-	-	-	-	-	-	-	-	-	-	*	*	-	-	-	-	-	-	-	-
Geranyl isovalerate[4]	-	-	-	-	-	-	-	-	-	-	-	-	-	-	-	-	-	-	-	-	-	-	-	-	-	-	-	*	*	-	-	-	-	-	-	-	17.40
Benzaldehyde	-	-	-	-	-	-	-	2.00	1.60	-	2.60	1.90	2.90	-	2.00	-	1.20	-	1.70	1.55	-	2.30	-	1.20	-	-	1.30	*	*	-	-	-	-	-	-	-	-
Monoterpeno Hidrocarboneto	6.06	2.03	18.51	32.62	23.73	9.76	0.70	0.10	6.02	0.25	12.67	7.98	0.30	-	-	7.45	6.73	5.75	11.78	-	0.20	3.40	9.20	1.20	0.50	0.25	6.72	*	*	30.70	28.90	21.20	21.40	21.40	35.60	16.70	16.60
Monoterpeno Oxigenado	-	-	-	32.08	27.79	-	5.83	2.07	2.78	4.00	3.76	3.80	3.60	1.33	2.84	3.66	3.18	2.42	3.30	2.00	3.48	2.84	2.72	3.90	4.64	5.26	2.28	*	*	-	-	-	-	-	16.30	31.10	24.20
Sesquiterpeno Hidrocarboneto	88.07	92.86	81.49	35.3	48.48	90.24	38.32	42.17	37.72	40.85	32.87	35.20	45.72	46.97	46.35	42.53	42.68	43.58	33.90	41.35	48.35	38.67	34.30	45.20	31.90	43.65	43.35	*	*	25.60	29.70	29.50	30.80	32.40	37.30	16.10	19.90
Sesquiterpeno Oxigenado	-	2.61	-	-	-	-	53.83	53.12	51.57	53.17	48.78	52.37	48.43	48.83	48.67	50.35	48.57	46.23	48.70	54.33	47.07	53.10	51.78	48.38	62.17	49.57	46.08	*	*	37.50	35.60	41.90	41.20	46.20	10.80	17.40	24.60

Legend: Cat = *P. cattleyanum*; Gua = *P. guajava*; Gui = *P. guineense*; Myr = *P. myrtoides*; Gau = *P. gaudichaudianum*; Fri = *P. friedrichsthalianum*; Psi = *Psidium* sp. The 5-mC% data of *P. guajava* accesses were revisited in the work published by our research group [1]. Data regarding the yield and content of the compounds identified in the

essential oil have been published by our research group for *P. guajava* [2,3], *P. guineense* [4] and *P. cattleyanum* [5]. *Chemical composition of *P. guineense* essential oil were not presented and considered in the correlation analysis.

References (ESM_3)

1. Alves, L. B., Noia, L. R., Canal, G. B., Ferreira, A. & Ferreira, M. F. da S. Epigenetic variation in guava (*Psidium guajava*) genotypes during the vegetative and reproductive phases of the production cycle. *Genet. Mol. Res.* **19**, (2020).

2. Mendes, L. A. *et al.* Spring alterations in the chromatographic profile of leaf essential oils of improved guava genotypes in Brazil. *Sci. Hortic. (Amsterdam).* **238**, 295–302 (2018).

3. de Souza, T. da S. *et al.* Chemotype diversity of *Psidium guajava* L. *Phytochemistry* **153**, 129–137 (2018).

4. Bernardes, C. de O. Diversidade genética, caracterização e atividade de óleos essenciais em *Psidium* spp. (Tese - Doutorado em Genética e Melhoramento) - Universidade Federal do Espírito Santo, Alegre-ES, 2017.

5. Spadeto, M. S. *et al.* Intraspecific C-value variation and the outcomes in *Psidium cattleyanum* Sabine essential oil. *Brazilian J. Biol.* **82**, 1–8 (2022).
